# Comparison of different methods of splenic hilar lymph node dissection for advanced upper- and/or middle-third gastric cancer

**DOI:** 10.1186/s12885-016-2814-z

**Published:** 2016-10-03

**Authors:** Xin Ji, Tao Fu, Zhao-De Bu, Ji Zhang, Xiao-Jiang Wu, Xiang-Long Zong, Zi-Yu Jia, Biao Fan, Yi-Nan Zhang, Jia-Fu Ji

**Affiliations:** Department of Gastrointestinal Surgery, Key laboratory of Carcinogenesis and Translational Research (Ministry of Education), Peking University Cancer Hospital & Institute, Haidian District Fucheng Road No. 52, Beijing, 100142 China

**Keywords:** Advanced gastric cancer, Splenic hilar lymph node dissection, Splenic preservation, Splenectomy

## Abstract

**Background:**

Surgery for advanced gastric cancer (AGC) often includes dissection of splenic hilar lymph nodes (SHLNs). This study compared the safety and effectiveness of different approaches to SHLN dissection for upper- and/or middle-third AGC.

**Methods:**

We retrospectively compared and analyzed clinicopathologic and follow-up data from a prospectively collected database at the Peking University Cancer Hospital. Patients were divided into three groups: in situ spleen-preserved, ex situ spleen-preserved and splenectomy.

**Results:**

We analyzed 217 patients with upper- and/or middle-third AGC who underwent R0 total or proximal gastrectomy with splenic hilar lymphadenectomy from January 2006 to December 2011, of whom 15.2 % (33/217) had metastatic SHLNs, and from whom 11.4 % (53/466) of the dissected SHLNs were metastatic. The number of harvested SHLNs per patient was higher in the ex situ group than in the in situ group (*P* = 0.017). Length of postoperative hospital stay was longer in the splenectomy group than in the in situ group (*P* = 0.002) or the ex situ group (*P* < 0.001). The splenectomy group also lost more blood volume (*P* = 0.007) and had a higher postoperative complication rate (*P* = 0.005) than the ex situ group. Kaplan–Meier (log rank test) analysis showed significant survival differences among the three groups (*P* = 0.018). Multivariate analysis showed operation duration (*P* = 0.043), blood loss volume (*P* = 0.046), neoadjuvant chemotherapy (*P* = 0.005), and N stage (*P* < 0.001) were independent prognostic factors for survival.

**Conclusions:**

The ex situ procedure was more effective for SHLN dissection than the in situ procedure without sacrificing safety, whereas splenectomy was not more effective, and was less safe. The SHLN dissection method was not an independent risk factor for survival in this study.

## Background

The estimated incidence and mortality of gastric cancer in 2013 were 984,000 and 841,000 worldwide, respectively [1, 2]. Globally, gastric cancer is the fifth most common cancer and the second most common cause of cancer death. More than 70 % of these cases occur in developing countries, with half arising in Eastern Asia (mainly Korea, Japan, and China). Surgery is the primary treatment for gastric cancer, with D2 lymphadenectomy widely accepted for advanced gastric cancer (AGC) in both Eastern and Western countries [3–5].

**Fig. 1:**
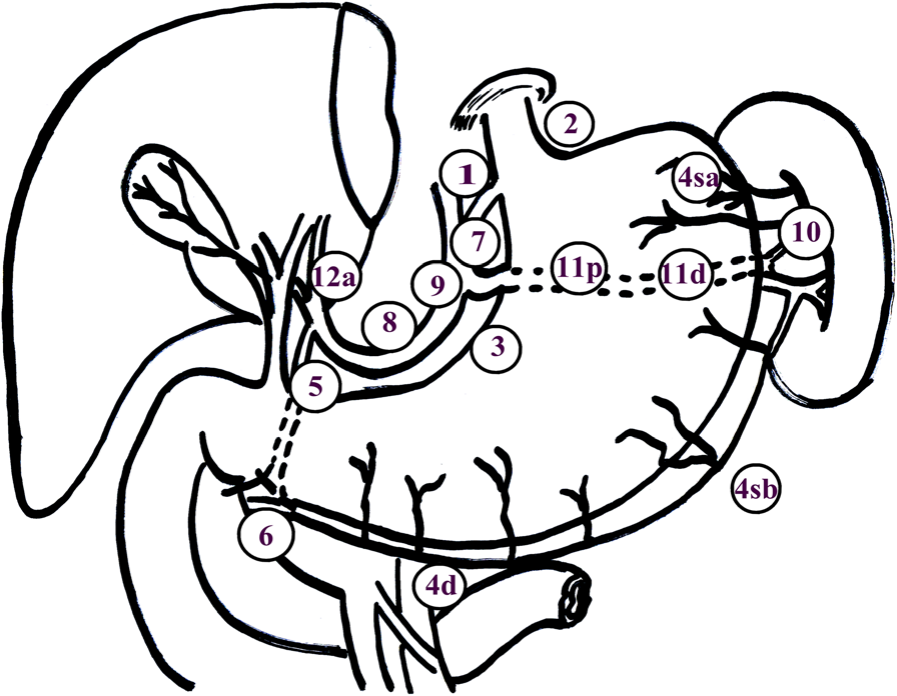
Definition of lymph node stations of gastric cancer. The lymph nodes of stomach are defined and given station numbers. Lymph node stations1-7, 8a, 9, 10, 11p, 11d and 12a are included in the D2 dissection for locally advanced upper and/or middle third gastric cancer

The incidence of upper- and/or middle-third gastric cancer has steadily increased, especially in Asia [6]. According to the 2010 Japanese gastric cancer treatment guideline (ver. 3) published by the Japanese Gastric Cancer Association, the extent of systematic lymphadenectomy depends on the type of gastrectomy [7]. The lymph node stations surrounding the stomach have been precisely defined by the Japanese Gastric Cancer Association (Table 1 and Fig. 1). To achieve sufficient negative proximal margins, most patients with upper- and/or middle-third AGC require total gastrectomies with D2 lymphadenectomies that include the splenic hilar lymph nodes (SHLNs; No. 10 lymph nodes) [8].

**Table 1:** Regional lymph nodes for gastric cancer

No	Definition
1	Right paracardial LNs
2	Left paracardial LNs
3a	Lesser curvature LNs along the branches of the left gastric artery
3b	Lesser curvature LNs along the 2nd branch and distal part of the right gastric artery
4sa	Left greater curvature LNs along the short gastric arteries
4sb	Left greater curvature LNs along the left gastroepiploic artery
4d	Left greater curvature LNs along the 2nd branch and distal part of the right gastroepiploic artery
5	Suprapyloric LNs along the 1st branch and proximal part of the right artery
6	Infrapyloric LNs along the 1st branch and proximal part of the right gastroepiploic artery
7	LNs along the trunk of left gastric artery between its root and the origin of tis ascending branch
8a	Anterosuperior LNs along the common hepatic artery
8p	Posterior LNs along the common hepatic artery
9	Celiac artery LNs
10	Splenic hilar LNs
11p	Proximal splenic artery LNs
11d	Distal splenic artery LNs
12a	Hepatoduodenal ligaments LNs along the proper hepatic artery
12p	Hepatoduodenal ligaments LNs along the portal vein
12b	Hepatoduodenal ligaments LNs along the bile duct

*LNs* lymph node

Reportedly, 7.3–26 % of SHLNs in upper- and/or middle-third AGC are metastatic [9–12]. Prophylactic splenectomy, in situ and ex situ spleen-preserving lymphadenectomies have been the most common dissection approaches for SHLNs. Prophylactic splenectomy was a common procedure for D2 dissection until the results of the Japanese Clinical Oncology Group (JCOG) 0110 study that showed a non-inferiority of spleen preservation compared with splenectomy in terms of overall survival [13, 14]. Nonetheless, as the JCOG 0110 study included only tumors from the lesser curvature, the approach for patients with tumors at the greater curvature is still in doubt.

Two main operative procedures for SHLN dissection spare the spleen. Ex situ and in situ dissection are defined depending on whether the pancreas and spleen are treated within the peritoneal cavity or not. The in situ dissection approach is more difficult as the SHLN dissection is implemented in a narrow and small space, and can thus lead to bleeding; however, it avoids moving the pancreas and spleen and shortens surgical time. In contrast, ex situ dissection is performed under direct vision, which provides a better exposure, and is thus less difficult.

To our knowledge, no previous study has directly compared the effectiveness and safety of these three approaches. We therefore investigated which of these three dissection approaches was better for patients with upper- and/or middle-third AGC.

## Methods

### Patients

This study was performed after approval by the Ethics Committee of Peking University Cancer Hospital. Informed consent was obtained from each patient. We retrospectively collected clinical and pathological data from a prospectively collected database at the Peking University Cancer Hospital. We included 217 patients with upper- and/or middle-third AGC who had undergone R0 total or proximal gastrectomy with SHLN dissection from January 2006 to December 2011. Their primary diagnoses were confirmed by endoscopic biopsies analysis. Clinical staging was mainly confirmed by ultrasound endoscopy, chest, abdominal and pelvic computed tomography scans, and laparoscopic exploration. Patients with other types of tumors, such as gastrointestinal stromal tumor or lymphoma, were excluded.

### Surgical procedure

All the enrolled patients underwent laparoscopic exploration to exclude distant metastatic disease. After that, all the patients received R0 resection with total or proximal gastrectomy and SHLN dissection. The lymph node dissection scope was mainly D2/D2+, according to the definition in the Japanese gastric cancer treatment guidelines [7]. The approach of SHLN dissection was at the discretion of the surgeon during the operation.

In the splenectomy group, splenectomy was performed with full mobilization of the distal pancreas and spleen. Lymph nodes along the splenic artery were completely dissected. The splenic artery was usually ligated and divided 5–6 cm away from its origin. The spleen and lymph nodes at the hilum of the spleen were removed, with the pancreas preserved.

In the in situ spleen-preserved group, the spleen and the pancreas were not mobilized from the retroperitoneum. Lymph nodes along the splenic artery were dissected. All the soft tissues at the splenic hilum were removed as cautiously as possible.

In the ex situ spleen-preserved group, splenic hilar lymphadenectomy was performed with full mobilization of the distal pancreas and spleen. The spleen was moved outside the peritoneal cavity. Lymph nodes along the splenic artery and at the splenic hilum were completely dissected, with the pancreas and spleen preserved, and then replaced into the peritoneal cavity.

After the surgery, the patients stayed in hospital to get recovery. Before they left the hospital, the discharge criteria must be all fulfilled. The discharge criteria included: absence of subjective complaints, tolerance of solid oral intake, return of bowel function, absence of intravenous fluids/medications, adequate mobility of daily living and self-care (eg, go to toilet, dress, shower, etc.), adequate pain control on oral analgesia only, adequate wound condition, removal of drainage tube, absence of infectious complications, absence of postoperative complications, absence of abnormal physical signs or laboratory test (eg, pulse, body temperature, white blood cell count, serum hemoglobin, etc.), acceptance of discharge, adequate home/social condition.

### Clinicopathologic parameters

The clinicopathological data collected from the database included age, sex, body mass index (BMI), neoadjuvant chemotherapy (NACT) regimens, tumor location, tumor size, presence of multi-tumor, range of gastrectomy, degree of lymph node dissection (LND), SHLN dissection procedure, tumor differentiation, lymphovascular invasion (LVI), depth of tumor invasion, number of harvested and metastatic lymph nodes, postoperative complications, mortality, length of postoperative hospital stay, operation duration, blood loss volume, and survival outcomes. Terminology used to describe the clinicopathologic parameters was based on the Japanese Gastric Cancer Association classification of gastric carcinoma [8].

### Follow-up

Follow-up was conducted mainly through telephone interviews, E-mail communication, or outpatient reviews. As of April 26, 2016, the percentage of follow-up was 96.7 % (210/217).

### Statistical analysis

All statistical analysis was performed through IBM SPSS Statistics 20.0 software (SPSS Inc., Armonk, NY). For quantitative variables, normal distribution was tested first. Variables of normal distribution were expressed as means ± standard deviation, and tested by analysis of variance among the three groups. If not, the variables were expressed as medians with ranges, and compared by Kruskal–Wallis non-parametric test. For categorical data, the chi-squared test or Fisher’s exact test was performed. Kaplan–Meier estimation and the log-rank tests were used to calculate survival. In the pairwise comparisons, the original calculated P value and the Bonferroni-corrected threshold were listed. If the P value was less than this Bonferroni-corrected threshold, then the comparison was considered to be statistically significant. Cox proportional hazards regression model was used to confirm independent prognostic factors through univariate and multivariate analysis. Except in the pairwise comparison, *P* < 0.05 (two-sided) was considered significant in the statistical analysis.

## Results

### Clinicopathologic parameters

We analyzed 217 patients in this retrospective study, who were divided into three groups: in situ (*n* = 68), ex situ (*n* = 118), and splenectomy (*n* = 31). Some of the patients in the splenectomy group had intended to undergo in situ or ex situ approach after abdominal exploration, but encountered unintended splenic injury resulting in splenectomy. Of all the thirty-one patients in the splenectomy group, two patients underwent conversion from in situ approach to splenectomy, and three patients underwent conversion from ex situ approach to splenectomy. The rates of conversion from in situ and ex situ procedures to splenectomy were 2.86 % (2/70) and 2.48 % (3/121), respectively. All of their clinicopathologic factors except the number of patients who received NACT and the range of gastrectomy were comparable among the three groups; however, lower percentages of the in situ group underwent NACT and total gastrectomies than the ex situ and splenectomy groups (Table 2).

**Table 2:** Patients’ clinicopathologic parameters

	In situ(*n* = 68), *n*(%)	Ex situ(*n* = 118), *n*(%)	Splenectomy(*n* = 31),*n*(%)	*P*value
Gender				0.238
Male	47(69.1)	91(77.1)	26(83.9)	
Female	21(30.9)	27(22.9)	5(16.1)	
Age				1.000
< 60	36(52.9)	63(53.4)	17(54.8)	
≥ 60	32(47.1)	55(46.6)	14(45.2)	
BMI				0.716
< 19	5(7.4)	10(8.5)	1(3.2)	
~ <25	46(67.6)	83(70.3)	22(71.0)	
~ <30	17(25)	22(18.6)	7(22.6)	
≥ 30	0(0)	3(2.5)	1(3.2)	
NACT				0.008
No	42(61.8)	48(40.7)	11(35.5)	
Yes	26(38.2)	70(59.3)	20(64.5)	
Degree of LND				0.090
D1+	5(7.4)	7(5.9)	1(3.2)	
D2	58(85.3)	84(71.2)	23(74.2)	
D2+	5(7.4)	27(22.9)	7(22.6)	
Gastrectomy				0.033
Proximal	28(41.2)	34(28.8)	5(16.1)	
Total	40(58.8)	84(71.2)	26(83.9)	
Differentiation				0.115
Well	1(1.5)	9(7.6)	5(16.1)	
Moderate	31(45.6)	53(44.1)	12(38.7)	
Poor	36(52.9)	57(48.3)	14(45.2)	
LVI				0.060
No	26(38.8)	66(55.9)	12(41.4)	
Yes	41(61.2)	52(44.1)	17(58.6)	
Location				0.648
EGJ	35(51.5)	63(53.4)	12(38.7)	
U/UM	7(10.3)	10(8.5)	6(19.4)	
M/MU	24(35.3)	43(36.4)	12(38.7)	
EUM	2(2.9)	2(1.7)	1(3.2)	
Tumor size				0.548
≤ 2 cm	7(10.3)	10(8.5)	2(6.5)	
~ ≤5 cm	35(51.5)	56(47.5)	10(32.2)	
~ ≤10 cm	21(30.9)	40(33.9)	14(45.2)	
> 10 cm	5(7.4)	12(10.2)	5(16.1)	
Multi-tumor				0.095
No	68(100)	115(97.5)	29(93.5)	
Yes	0	3(2.5)	2(6.5)	
T stage^a^				0.059
T2	5(7.4)	11(9.3)	2(6.5)	
T3	5(7.4)	1(0.8)	1(3.2)	
T4a	51(75.0)	101(85.6)	23(74.2)	
T4b	7(10.3)	5(4.2)	5(16.1)	
N stage^a^				0.230
N0	8(11.8)	35(29.7)	5(16.1)	
N1	13(19.1)	16(13.6)	7(22.6)	
N2	16(23.5)	26(22.0)	6(19.4)	
N3a	18(26.5)	21(17.8)	8(25.8)	
N3b	13(19.1)	20(16.9)	5(16.1)	

*BMI* body mass index, *NACT* neoadjuvant chemotherapy, *LND* lymph node dissection, *LVI* lymphovascular invasion, *EGJ* esophagogastric junction, *E* esophagus, *U* upper, *M* middle

^a^7th UICC/AJCC TNM classification for gastric cancer

### Splenic hilar lymphadenectomy

All 217 patients in our study underwent SHLN dissection, and all of the dissected lymph nodes were confirmed by pathological examination. Of the 217 patients, 33 (15.2 %) were found to have metastatic SHLNs, including 8.8 % (6/68) of the in situ group, 14.4 % (17/118) of the ex situ group, and 32.3 % (10/31) of the splenectomy group (*P* = 0.010). Of 466 harvested SHLNs, 11.4 % (53/466) were metastatic, including 8.3 % (10/121) in the in situ group, 11.8 % (32/271) in the ex situ group, and 14.9 % (11/74) in the splenectomy group (*P* = 0.349).

### Intraoperative and postoperative parameters

Surgery-related parameters were compared among the three groups (Tables 3 and 4), and were found to differ significantly in the number of harvested SHLNs per patient (*P* = 0.047), length of postoperative hospital stay (*P* = 0.001), and blood loss volume (*P* = 0.027). Further paired comparisons revealed that the number of harvested SHLNs per patient was higher in the ex situ group than in the in situ group (*P* = 0.015). The length of postoperative hospital stay was significantly longer in the splenectomy group than in the other two groups (splenectomy vs in situ: *P* = 0.002; splenectomy vs ex situ: *P* < 0.001). The splenectomy group also had significantly greater blood loss volume than did the ex situ group (*P* = 0.007). The three groups did not significantly differ in total harvested lymph nodes per patient (*P* = 0.313) or operation duration (*P* = 0.695).

**Table 3:** Patients’ intraoperative and postoperative parameters

	In situ (*n* = 68)	Ex situ (*n* = 118)	Splenectomy (*n* = 31)	*P*value
No. of harvested SHLNs, median (range)	1(1–4)	2(1–7)	2(1–7)	0.047
No. of total harvested LNs, median (range)	33(10–66)	33(11–78)	31(11–60)	0.313
Postoperative hospital stay, days, (mean ± standard deviation)	16.41 ± 3.06	15.11 ± 1.53	23.26 ± 4.74	0.001
Blood loss volume, ml, (mean ± standard deviation)	211.62 ± 53.43	180.08 ± 24.71	262.90 ± 78.09	0.027
Operation duration, min, (mean ± standard deviation)	242.66 ± 18.90	244.24 ± 13.66	247.65 ± 22.06	0.695
Postoperative complication rate, *n* (%)	12(17.6 %)	15(12.7 %)	11(35.5 %)	0.011
Bleeding, *n* (%)	1 (1.5 %)	3 (2.5 %)	3 (9.7 %)	0.153
Anastomotic leak, *n* (%)	3 (4.4 %)	6 (5.1 %)	5 (16.1 %)	0.111
Pancreatic fistula, *n* (%)	0 (0.0 %)	0 (0.0 %)	2 (6.5 %)	0.019
Abdominal effusion, *n* (%)	6 (8.8 %)	7 (5.9 %)	5 (16.1 %)	0.183
Abdominal infection, *n* (%)	6 (8.8 %)	8 (6.8 %)	7(22.6 %)	0.029
Pneumonia, *n* (%)	3 (8.8 %)	1 (0.8 %)	1(3.2 %)	0.269
Reoperation rate, *n* (%)	3(4.4 %)	3(2.5 %)	2(6.5 %)	0.359
Mortality rate, *n* (%)	0(0 %)	1(0.8 %)	1(3.2 %)	0.363

*SHLNs* splenic hilar lymph nodes, *LNs* lymph nodes

**Table 4:** Pairwise comparisons of operative parameters and morbidity

	*P*value*		
	In situ vs splenectomy	In situ vs Ex situ	Splenectomy vs Ex situ
No. of harvested SHLNs	0.154	0.015	0.755
Postoperative hospital stay	0.002	0.832	<0.001
Blood loss volume	0.058	0.388	0.007
Postoperative complication rate	0.047	0.239	0.005

*SHLNs* splenic hilar lymph nodes, *LNs* lymph nodes

*Bonferroni correction was carried out. *P* < 0.017 (two-sided) was considered significant

Postoperative complication rates were: in situ group: 17.6 % (12/68); ex situ group: 12.7 % (15/118); and splenectomy group: 35.5 % (11/31; *P* = 0.011); and were notably higher in the splenectomy group than in the ex situ group (*P* = 0.005; paired comparison). The three groups did not significantly differ in reoperation rate (*P* = 0.359) or postoperative mortality rate (*P* = 0.363).

### Survival outcomes

**Fig. 2:**
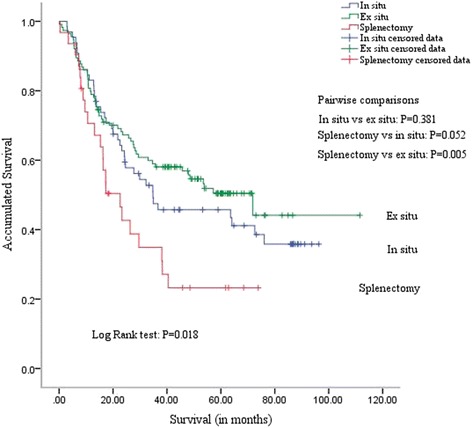
Survival curves for three groups. The ex vivo, in vivo, and splenectomy groups significantly differed in survival (*P* = 0.018, log-rank test). This difference was especially pronounced between the ex situ and splenectomy groups (*P* = 0.005, *P* < 0.017). In the pairwise comparisons, Bonferroni modification was carried out. *P* < 0.017 (two-sided) was considered significant

As of April 26, 2016, median follow-up time was 33.2 months (range: 1–111 months). Median survival times were: in situ group: 34.5 months, ex situ group: 71.1 months, and splenectomy group: 21.1 months; 5-year overall survival rates were: in situ group: 46 %, ex situ group: 50 %, and splenectomy group: 23 %. The three groups were found to significantly differ by Kaplan–Meier survival analysis (log rank test; *P* = 0.018; Fig. 2), especially the splenectomy and ex situ groups (*P* = 0.005; paired comparisons; Fig. 2).

Risk factors found in the univariate analysis included SHLN dissection method, operation duration, blood loss volume, postoperative complications, use of NACT, presence of multiple tumors, differentiation, tumor size, T stage, N stage, LVI, and range of gastrectomy (Table 5). All these factors were subjected to multivariate analysis, which found operation duration (*P* = 0.043), blood loss volume (*P* = 0.046), use of NACT (*P* = 0.005), and N stage (*P* < 0.001) to be independent prognostic factors for survival (Table 5).

**Table 5:** Univariate and multivariate analysis of prognostic factors

	Univariate HR (95 % CI)	*P*value	Multivariate HR (95 % CI)	*P*value
Gender		0.679		
Male	1			
Female	0.909(0.580,1.427)			
Age		0.254		
< 60	1			
≥ 60	1.238(0.857,1.788)			
BMI		0.287		
< 19	1			
~ <25	0.578(0.307,1.090)	0.090		
~ <30	0.496(0.241,1.020)	0.057		
≥ 30	0.635(0.140,2.873)	0.555		
Postoperative hospital stay	1.010(0.995,1.026)	0.205		
Reoperation		0.899		
No	1			
Yes	1.067(0.393,2.896)			
Location		0.100		
EGJ	1			
U/UM	0.991(0.519,1.895)	0.979		
MU/M	1.394(0.937,2.074)	0.101		
EUM	2.908(1.048,8.071)	0.040		
Degree of LND		0.701		
D1+	1			
D2	1.286(0.562,2.942)	0.551		
D2+	1.082(0.426,2.747)	0.868		
SHLN dissection method		0.020		0.061
In situ	1		1	
Ex situ	0.822(0.541,1.249)	0.369	0.804(0.510,1.269)	0.349
Splenectomy	1.671(0.983,2.840)	0.058	1.522(0.865,2.678)	0.145
Operation duration	1.005(1.003,1.007)	<0.001	1.003(1.000,1.005)	0.043
Blood loss volume	1.001(1.001,1.002)	<0.001	1.001(1.000,1.002)	0.046
Postoperative complications		0.036		0.468
No	1		1	
Yes	1.607(1.030,2.507)		1.210(0.723,2.027)	
NACT		0.007		0.005
No	1		1	
Yes	1.689(1.157,2.466)		2.289(1.501,3.492)	
Multi-tumor		0.011		0.099
No	1		1	
Yes	3.203(1.301,7.887)		2.402(0.849,6.800)	
Differentiation		0.039		0.319
Well	1		1	
Moderate	1.200(0.512,2.816)	0.674	1.155(0.474,2.818)	0.751
Poor	1.886(0.816,4.361)	0.138	1.550(0.643,3.734)	0.329
Tumor size		0.007		0.135
≤ 2 cm	1		1	
~ ≤5 cm	3.075(1.110,8.524)	0.031	2.931(0.877,9.794)	0.081
~ ≤10 cm	4.227(1.520,11.754)	0.006	3.420(0.989,11.828)	0.052
> 10 cm	5.702(1.888,17.220)	0.002	4.710(1.254,17.684)	0.022
T stage^a^		0.040		0.572
Serosa negative	1		1	
T4a	1.728(0.872,3.428)	0.117	1.541(0.651,3.652)	0.326
T4b	3.036(1.275,7.227)	0.012	1.645(0.604,4.482)	0.330
N stage^a^		<0.001		<0.001
N0	1		1	
N1	1.482(0.755,2.908)	0.253	1.398(0.696,2.808)	0.347
N2	1.937(1.308,3.616)	0.038	1.836(0.933,3.609)	0.078
N3a	1.714(0.904,3.250)	0.099	1.958(0.988,3.883)	0.054
N3b	5.441(2.950,10.033)	<0.001	6.327(3.181,12.582)	<0.001
LVI		0.004		0.334
No	1		1	
Yes	1.727(1.182,2.523)		1.299(0.764,2.209)	
Gastrectomy		0.001		0.203
Proximal	1		1	
Total	2.098(1.336,3.224)		1.368(0.844,2.218)	

*BMI* body mass index, *EGJ* esophagogastric junction, *E* esophagus, *U* upper, *M* middle, *LND* lymph node dissection, *SHLN* splenic hilar lymph node, *NACT* neoadjuvant chemotherapy, *LVI* lymphovascular invasion

^a^7th UICC/AJCC TNM classification for gastric cancer

## Discussion

Although the incidence of gastric cancer has decreased worldwide, upper- and/or middle-third AGC has shown an increasing trend. As far as we know, the only way to cure gastric cancer is radical surgery, which includes gastrectomy and lymph node dissection. The currently recommended surgical procedure for advanced upper- and/or middle-third gastric cancer is total gastrectomy with D2 lymph node dissection [7]. SHLNs are defined as group No.10 lymph nodes, which are included in D2 dissection. Reportedly, the incidence of SHLN metastasis in the upper- and/or middle-third AGC is 7.3–26 %, which was higher than in the lower-third gastric cancers [9–12, 15–17]. In our study, the incidence of metastasis of SHLNs was 15.2 % (33/217), which was similar to previous reported studies, while the rates in the in situ, ex situ, and splenectomy groups were 8.8 % (6/68), 14.4 % (17/118), and 32.3 % (10/31), respectively (*P* = 0.010). Surgeons were inclined to use ex situ or splenectomy procedures for SHLNs suspected of having metastases, to perform dissections more effectively.

Optimal procedure for SHLN dissection has long been debated. Many previous studies have reported that splenectomy in this situation did not lead to longer survival [12, 18, 19], and in fact might increase surgical complication and mortality rate. During splenectomy, the pancreas tail and spleen are mobilized, which often leads to pancreatic fistulae or abscess formation. Moreover, loss of the spleen and its effect on immune function might adversely affect the recovery process. In 2016, the JCOG 0110 study reported that prophylactic splenectomy should be avoided for both surgical safety and survival benefit in total gastrectomies for proximal gastric cancers that do not invade the greater curvature [14]. Although patients whose cancers involved the greater curvature were not included in the JCOG 0110 study, it was the largest randomized clinical trial of splenectomy in gastric cancer, and demonstrated significant non-inferiority of spleen preservation for the first time. In our study, splenectomy reduced surgical safety and slowed the speed of postoperative recovery in terms of operative blood loss volume and postoperative hospital stay, compared with the spleen-sparing procedures. Our splenectomy group had longer average postoperative hospital stay, higher average blood loss volume, and a higher postoperative complication rate than the ex situ group, which was in accordance with earlier studies [19–21].

We found ex situ procedure was more effective for SHLN dissection than in situ splenic-preserving procedure and did not sacrifice surgical safety. Ex situ spleen-preserving procedure might improve the integrity of lymphadenectomy at the splenic hilum [22]. In the ex situ group, dissection of SHLNs was conducted under direct vision, and allowed surgeons to protect blood vessels and clear fatty tissues at the splenic hilum much more easily than in the in situ group, where dissection of SHLNs was very difficult and injury to spleen and blood vessels sometimes occurred. Therefore, although more time was required to mobilize the spleen and pancreas tail, the time needed to dissect SHLNs was significantly reduced. For this reason, operation duration was comparable between the in situ and ex situ groups. In our study, the ex situ procedure was more effective, and did not increase operation duration.

Interestingly, although Kaplan–Meier and log-rank analysis showed significant differences in survival among the three groups, Cox regression analysis of proportional hazards did not show SHLN dissection procedure to be an independent risk factor for survival. The significant difference shown in the Kaplan–Meier method might be caused by some other factors such as the imbalance of grouping in our study. The higher postoperative complication rate in the splenectomy group probably had adverse effects on survival, which is supported by earlier studies [23, 24].

Our study also had some limitations. First, it was a retrospective study, and selection bias was difficult to avoid. For instance, the percentages of patients who received NACT were much higher in the splenectomy and ex situ groups, probably because patients with later-stage disease were more likely to receive NACT. The choice of lymphadenectomy procedure was decided by surgeons, who usually chose patients with later-stage disease for ex situ or splenectomy procedures, as these methods seem to be more effective means to dissect the SHLNs. Similarly, more patients in the splenectomy or ex situ groups underwent total gastrectomies, which are more suitable for patients with later-stage disease. The three groups did not significantly differ with regard to other clinicopathologic parameters. Second, as the sample size in the splenectomy group was much smaller than that in the other two groups, a type II error might have occurred.

## Conclusions

Ex situ SHLN dissections were safer than splenectomies. Compared with in situ procedures, ex situ procedures apparently dissected SHLNs more effectively. Although the survival in these three groups significantly differed in Kaplan–Meier analysis, SHLN dissection method was not an independent risk factor for survival. Multicenter, large-scaled, randomized controlled trials are needed to clarify the optimal splenic hilar lymphadenectomy procedure.

## Abbreviations

AGC: Advanced gastric cancer
BMI: Body mass index
JCOG: Japanese Clinical Oncology Group
LND: Lymph node dissection
LVI: Lymphovascular invasion
NACT: Neoadjuvant chemotherapy
SHLN: Splenic hilar lymph node (No. 10 lymph node)
